# The effect of LyPRP/collagen composite hydrogel on osteogenic differentiation of rBMSCs

**DOI:** 10.1093/rb/rbaa053

**Published:** 2020-12-11

**Authors:** Manyu Chen, Quanying Liu, Yang Xu, Yuxiang Wang, Xiaowen Han, Zhe Wang, Jie Liang, Yong Sun, Yujiang Fan, Xingdong Zhang

**Affiliations:** 1 National Engineering Research Center for Biomaterials, Sichuan University, 29 Wangjiang Road, Chengdu, Sichuan 610064, P. R. China; 2 Department of Medical Genetics, Zunyi Medical University, No. 6 West Xuefu Road, Zunyi, Guizhou 563000, P. R. China; 3 Sichuan Testing Center for Biomaterials and Medical Devices, Sichuan University, 29 Wangjiang Road, Chengdu 610064, P. R. China

**Keywords:** platelet-rich plasma, freeze-dried platelet-rich plasma, collagen hydrogel, osteogenic differentiation

## Abstract

Although platelet-rich plasma (PRP) plays a significant role in the orthopedic clinical application, it still faces two major problems, namely, uncontrollable factors release, frequent preparation and extraction processes as well as the inconvenient form of usage. To overcome these shortcomings, freeze-dried PRP (LyPRP) was encapsulated into bioactive Col I hydrogel to induce osteogenic differentiation of rabbit bone marrow mesenchymal stem cells (rBMSCs). And PRP/Col І composite hydrogel was prepared as a control. Compared with Col І hydrogel, the introduction of platelets significantly improved the mechanical properties of hydrogels. Meanwhile, platelets were evenly distributed in the composite hydrogels network. The sustainable release of related factors in the composite hydrogels could last for more than 14 days to maintain its long-term biological activity. Further cell experiments confirmed that PRP and LyPRP could effectively alleviate the contraction of collagen hydrogel *in vitro*, and promote the adhesion, proliferation and osteogenesis differentiation of rBMSCs. The results of osteogenic gene expression indicated that the 10% LyPRP/Col І composite hydrogel could facilitate the early expression of BMP-2 and late osteogenic associated protein formation with higher expression of alkaline phosphatase and Osteocalcin (OCN). These results might provide new insights for the clinical application of 10% LyPRP/Col І composite hydrogel as practical bone repair injection.

## Introduction

As a core component of tissue engineering, growth factors play a key role in regulating growth and metabolism, and have a vital impact on bone regeneration [1]. Platelets, containing various growth factors, are the main regulators in the inflammatory phase and play a crucial role in the growth and differentiation processes [2, 3]. Autologous plasma has been separated, concentrated and mixed with thrombin to produce platelets products with growth factor-rich for bone repair at injury site [4−7], which was prepared *in vitro* and was defined as platelet-rich plasma (PRP). In 1998, Dr Robert E. Marx first proposed the use of PRP to enhance the initial stage of bone wound healing [8]. Since then, PRP had been widely used in pre-clinical and clinical applications for bone regeneration. Previous studies had shown that PRP could accelerate bone defects repair by promoting cell proliferation and differentiation in the early stage [9, 10]. Although its action mechanism is still unclear, experiences have shown that autologous PRP can treat bone injury. The immediate reason for the clinical application of PRP is that a large number of autologous growth factors are released from platelets after activated by the activator. Among them, transforming growth factor-β (TGF-β), vascular endothelial growth factor (VEGF) and platelet-derived growth factor (PDGF-AB) have the highest concentration, which can promote bone repair by stimulating collagen synthesis, callus formation and tissue regeneration [11, 12].

However, two major issues greatly limited its application. The first problem was that the short biological activity of growth factors *in vivo*. To solve this problem, the combination of bio-scaffold delivery had been extensively studied. Proper bio-scaffold could directly accelerate the healing or regeneration of bone tissue [13, 14]. However, the uncontrolled bioactive substances delivery would lead to undesirable tissue repair effect. For example, the high concentration of TGF-β was proved to be a harmful substance. Therefore, it must be reasonable that bio-scaffold could effectively and efficiently deliver bioactive substances without an initial burst effect. By extending the release period of growth factors, PRP could also promote bone repair in the middle and late stages. The second problem was the immediate preparation of PRP from the patient’s peripheral blood during every application process. Meanwhile, there was no long-term (>1 week) method for PRP preservation, which limited the application of PRP. Platelets are very flimsy to some extent, with an average life span of 7–11 days, and are easily activated after leaving the physiological environment, which will lead to the loss of their original biological activity [15]. To solve this problem, it is recommended to store PRP as freeze-dried powders (LyPRP) for preservation and application with a long lifetime.

Type I collagen (Col I) was a major organic component of bone tissue, which had a good biological activity for promoting cell adhesion and proliferation [16]. However, the relatively low osteogenic bioactivity and mechanical properties limited to its wide application [17]. To overcome these limitations as described above, collagen hydrogels was chosen as gradual release carrier for growth factors to investigate osteogenic differences between PRP and LyPRP with different concentrations. The physical and chemical properties of composite hydrogels, the distribution of biologically active ingredients, and the release behavior of growth factors were evaluated. Meanwhile, the proliferation, adhesion and osteogenic differentiation of encapsulated rabbit bone marrow mesenchymal stem cells (rBMSCs) in composite hydrogels were explored. Based on above results, the 10% PRP/Col I and 10% LyPRP/Col I hydrogels presented better osteogenic differentiation ability. Especially for LyPRP/Col I hydrogel, it could realize the long-term release of active factors and accelerate osteogenic differentiation in the later stage of treatment. These works would provide more pragmatic insights into the design of clinically applicable bone repair scaffolds.

## Materials and methods

### Materials

Type I collagen was extracted and purified from new-born calfskin in our laboratory [18]. Ice acetic acid (CH_3_COOH), Silica gel (500 G, DOW CORNING), PGE1 (Powder, Beijing Huazhong Haiwei Co., LTD) was dissolved into ultrapure water to prepare a 50 mM stock solution, DMEM medium, α-Minimum Essential Medium (α-MEM, Hyclone, USA), Phosphate Buffered Saline (PBS, 0.0067 M) and Antibiotics (1×, 100 ml, Hyclone) were bought from Thermo Fisher Scientific Corporation (USA). Fetal bovine serum (FBS, Gibco, Australia origin) was obtained from Life Technologies Corporation (USA).

### Preparation and characterization of PRP

All animal procedures were performed in accordance with the guidelines for care and use of Laboratory Animals of Sichuan University. Fresh blood samples were obtained from New Zealand white rabbits weighing 2.5 − 3.0 kg. Peripheral venous blood was drawn from the central auricular artery of a rabbit using a 5 ml sterile blood collection tube containing heparin sodium as an anticoagulant. PRP was prepared by a two-step centrifugation process. Briefly, most red blood cell in whole blood was discarded by first centrifuging at 2000 rpm for 10 min at room temperature. And then, the solution containing platelets and white blood cells was centrifuged again at same condition. Finally, PRP was harvested by removing the supernatant. Generally, the 1 ml PRP was harvested from 10 ml whole blood. The blood cell counts of PRP samples were analyzed by automated blood cell counter.

### Preparation of LyPRP

The freeze-dried PRP pretreatment solution (pH = 6.7) was prepared as described in literature [19]. To prevent platelets from being activated prematurely, platelet activation inhibitor PGE1 (1 μl/ml) was added to the above solution. The lyophilizing solution (pH = 6.7) was prepared by mixing 30% platelet-poor plasma supernatant with the above-mentioned LyPRP pretreatment solution. The whole blood was collected by aforementioned method, then centrifuged at 2000 rpm for 10 min. Removing the upper layer plasma, the same volume of LyPRP pretreatment liquid was added as the removed liquid to the remaining solution. The liquid in the centrifuge tube was evenly blown, then placed in a 37°C water bath and shaken for 2 − 4 h, which allowed the solution in the tube to be thoroughly mixed and also made the platelets contained in the solution evenly dispersed. After the water bath was completed, the solution containing suspended platelets was centrifuged again. After centrifugation, the supernatant was carefully discarded. Besides, the same volume as the discarded supernatant, the prepared lyophilizing solution was added to remaining solution. The final liquid was freeze-dried. Finally, the LyPRP powder was prepared. The blood cell counts of LyPRP samples were analyzed by automated blood cell counter.

### Preparation of PRP/col I and LyPRP/col I composite hydrogels

The pH of the collagen solution (2 wt%) was adjusted to about 7 with 1 M NaOH solution. The prepared neutral collagen gel was added with different concentrations (5, 10 and 20%) of PRP and LyPRP solution, and mixed into a homogeneous solution. In this hybrid solution, the final concentration of the collagen solution was 7 mg/ml. The following step was to transfer the composite hydrogels to the self-made silicone molds immediately (diameter 7 mm, height 2 mm). Next, these molds were placed in 37°C environment, and waited for 5 − 15 min for forming gels.

### Release behavior of growth factors in PRP/col I and LyPRP/col I composite hydrogels

The prepared composite hydrogels were placed in 4 ml centrifuge tubes, in which was added 2 ml α-MEM medium. The extracts were collected in 1, 3, 7 and 14 days, respectively. Meanwhile, the same volume fresh culture medium as extracts was added to the corresponding centrifuge tubes. The collected extracts were placed at −80°C. After all the samples of the extracted liquid were collected at all time points, the release of related growth factors (TGF-β, VEGF and PDGF-AB) in the extraction liquid was detected by enzyme-linked immunosorbent assay kit (ELISA).

### Morphology characterization of composite hydrogels

The internal microstructure of the composite collagen was observed under the scanning electron microscope (SEM, HITACHI S-800, Japan). The hydrogel samples were immersed in the 2.5% glutaraldehyde solution at room temperature to fix for 24 h and were washed with PBS for three times. Each wash takes 5 min. After the wash step, the fixative was washed away thoroughly. Then, the next procedure was placed the composite hydrogels in 20, 40, 60, 80 and 100% ethanol successively for gradient dehydration. Each dehydration was carried out for 15 min. After dehydration, the surface of the composite hydrogels was sprayed with a thin layer of gold and dried at the critical point, followed by scanning electron microscopy. The size analysis of platelets in hydrogels was measured by using ImageJ software (National Institutes of Health, Bethesda, USA).

### The mechanical property of composite hydrogels

The storage modulus (*G*′) and loss modulus (*G*″) of the different concentrations composite hydrogels were determined by a Dynamic Mechanical Analyzer (DMA, TA-Q800, USA) instrument in the multi-frequency mode with 1, 2 and 5 Hz at room temperature. The testing parameters were set at an amplitude of 20 μm, a preload force of 0.002 N and a force track of 105%. Every sample was measured in triplicate.

### The degradation performance of composite hydrogels

The different contents of PRP and LyPRP hybrid hydrogels were freeze-dried and weighed (Wo), and then soaked in PBS and incubated in a 37°C constant temperature shaker (ZHWY-2012C, Shanghai Zhicheng, China), shake speed at 90 rpm. At certain time intervals, the hydrogels were taken out, freeze-dried and weighed (Wr). All results were estimated from the data of three individual experiments. The degradation of the composite hydrogel was expressed as a percentage of weight loss. Degradation rate (%) = (Wo−Wr)/Wo × 100%

### Proliferation and size morphology of composite hydrogels encapsulated rBMSCs

rBMSCs were extracted from rabbit bone marrow [20], primary cells were maintained in α-MEM medium (Hyclone) and supplemented with 20% FBS (Gibco, USA) and 1% penicillin/streptomycin (PS, Gibco). When the cultured cell fusion grows to cover 90% of the entire area of the culture dish, the cells can be passaged. At the moment, α-MEM medium containing 10% FBS was used to culture the cells were passaged. In this experiment, cells passaged to the third generation can be used. The pH of the collagen solution (2 wt%) was adjusted to about 7 with 1 M NaOH solution. The prepared neutral collagen gel was added with rBMSCs cell suspension and different concentrations of PRP and LyPRP solution to make the final cell concentration reach to 3 × 10^6^ cells/ml [21]. The composite hydrogels blended with rBMSCs were injected into the above-mentioned self-made silicone molds and incubated at 37°C until the gels formed. Each ultra-low viscosity 24-well plate (Costar) was placed a hydrogel block and added 1.5 ml of culture medium. All samples were cultured under the same conditions (5% CO_2_, 37°C) in a cell incubator.

After hybrid hydrogels encapsulated rBMSCs were cultured for 1, 3, 7 and 14 days *in vitro*, the detection of Cell Counting Kit-8 (CCK-8) and Live/Dead staining by fluorescein diacetate (FDA, live, green) and propidium iodide (PI, dead, red) were carried out to detect cell attachment and proliferation ability. The cytoskeletal F-actin staining was performed using rhodamine-phalloidin (red) and DAPI (blue) and observed by a confocal laser scanning microscope. Briefly, after culturing for 1, 3, 7 and 14 days, the hydrogels were washed with PBS for three times, and incubated with 10% CCK-8 solution for 4 h in a 37°C incubator. In addition, without any hydrogel samples also were added with CCK-8 solution as a control group. The absorbance value at 450 nm was measured by using a Thermo Scientific Varioskan Flash Multiscan Spectrum. All absorbance values were expressed relative to no sample control.

After hybrid hydrogels encapsulated rBMSCs were cultured for 1, 3, 7 and 14 days *in vitro*, the original medium was removed. The hydrogels were washed twice with PBS and immersed in PBS solution containing 1 µg/ml of FDA and 1 µg/ml of PI, which stained viable cells and dead cells, respectively, and then washed with PBS solution again for 1 min.

In order to explore the effect of PRP and LyPRP composite collagen scaffold wrapped rBMSCs on collagen contraction. After different composite hydrogels were cultured in the medium for 1, 3, 7 and 14 days, samples were collected and taken photos with a camera. The size analysis of hydrogel was measured by using ImageJ software (National Institutes of Health). Every sample was measured in triplicate.

### Osteo-differentiation of rBMSCs encapsulated in composite hydrogels

For the osteo-differentiation experiments, all hydrogel groups encapsulated rBMSCs were cultured in high-glucose DMEM supplemented with 10% FBS, 1% PS, 0.008 µg/ml β-glycerol phosphate , 1.76 µg/ml ascorbic acid and 0.66 µg/ml dexamethasone (Sigma-Aldrich). For sections staining, these hydrogels were cultured for 3, 7, 14 and 21 days *in vitro*, and washed twice with PBS solution, and soaked in 4% paraformaldehyde solution for 48 h. The samples were processed for the paraffin section, and the sections (10 µm) were processed for hematoxylin-eosin (H&E), Alizarin red S (ARS) histological staining and alkaline phosphatase (ALP) and osteopontin (OPN) immunohistochemical (IHC) staining (Servicebio, China).

For quantitative real-time polymerase chain reaction (q-PCR) analysis, these hydrogels were cultured for 7, 14 and 21 days *in vitro* were collected by RNA pyrolysis liquid and stored at −80°C until to use. The TRIzol reagent (Qiagen, Germany) was used to extract the total cellular RNA of the cells. The RNA reverse-transcribed into cDNA using the cDNA Synthesis Kit (Bio-Rad, USA). The targeted genes were detected by SYBR GREEN I real-time PCR (Bio-Rad). Primers (5′−3′) used in this study were listed in Supplementary Table S1 in the supporting information. The internal control for normalization was the GAPDH gene (glyceraldehyde 3-phosphate dehydrogenase). Before performing the expression of each gene, the internal reference gene GAPDH in each sample should be detected, and the concentration of reverse transcribed RNA (cDNA) should be adjusted according to the results of the internal reference gene. The entire qPCR reaction system (10 µl) includes upstream primer (1 µl), downstream primer (1 µl), RNase-free water (2 µl), Scofast EraGreen Supermix (5 µl), cDNA (1 µl). The samples to be tested were mixed and put into a real-time fluorescent PCR quantitative system to detect the expression of related genes. The relative expression level of the sample is calculated by the 2^−△△Ct^ method (Table 1).

**Table 1.** Specific primers for PCR

Gene	Primers
ALP	Forward: 5′-ATGACAACCAGAATGAGCCAACG-3′ Reverse: 5′-CACCGACAATGCCACTTCCAC-3′
BMP2	Forward: 5′-ACCATGGGTTTGTGGTGGAA-3′ Reverse: 5′-CCGCTGTTTGTGTTTCGCTT-3′
OCN	Forward: 5′-CTCACTCTTGTCGCCCTGCT-3′ Reverse: 5′-CTCTTGGACACGAAGGCTGAG-3′
GAPDH	Forward: 5′-CCGCCCAGAACATCATCCCT-3′ Reverse: 5′-GCACTGTTGAAGTCGCAGGAGA-3′

### Statistical analysis

Results were presented as mean ± standard deviation (SD) of three representative experiments. Statistical analysis was performed using a one-way analysis of variance followed by Tukey’s post hoc testing using SPSS 22.0 software. The significance level was set at *P* < 0.05 (*), *P* < 0.01 (**), *P* < 0.001 (***).

## Results and discussion

### Analysis of blood cell components in PRP and LyPRP

**Figure 1.**
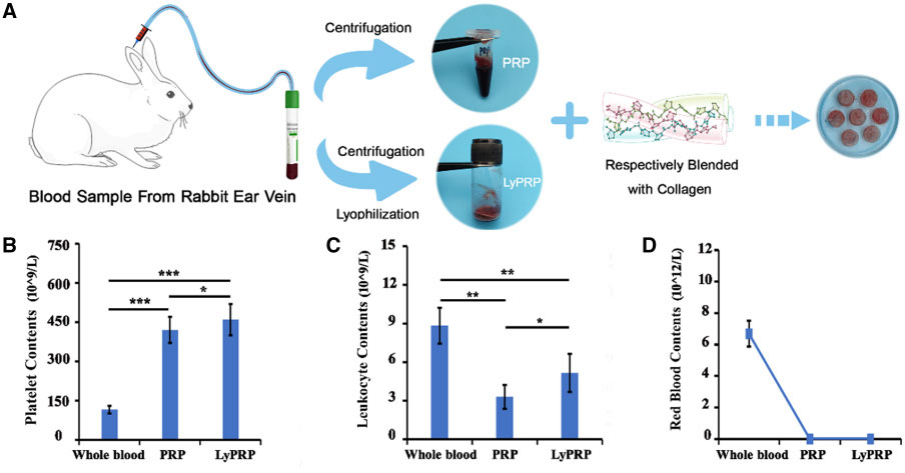
(**A**) Schematic illustration showing the fabrication process of composite hydrogels. Blood cell counts of whole blood, PRP and LyPRP by an automated blood cell counter. (**B**) Platelet. (**C**) Leukocyte. (**D**) Red blood cell. The data were presented as mean ± SD from three independent experiments (*n* = 3). ∗*P* < 0.05, ∗∗*P* < 0.01 and ∗∗∗*P* < 0.001.

The preparation process of PRP and LyPRP was shown in Fig. 1A. And the contents of related platelets, white blood cells and red blood cells were measured separately. Figure 1B shows that the platelet content in PRP and LyPRP were about four times that of whole blood, which reached the effective enrichment concentration as mentioned in the literature [22]. But the platelet content in LyPRP was higher than that in PRP. The results in Fig. 1C show that compared with whole blood, significantly lower white blood cells existed in PRP and LyPRP groups, indicating that the extracted PRP by this method was poor leukocyte PRP. Literature had reported that a high concentration of white blood cells might have an adverse effect on bone repair by releasing inflammatory factors (such as IL-1β, TNF-α), thereby affecting or even counteracting the promoting effect of growth factors on the bone repair [23]. Analysis by hemocytometer showed that almost no red blood cells were found in PRP and LyPRP (Fig. 1D). The above results indicated that compared with PRP, the lyophilization method maintained the concentration of platelets well and presented the same effect in inhibiting white blood cell residue.

### Characteristics of PRP/Col I and LyPRP/Col I composite hydrogels

**Figure 2.**
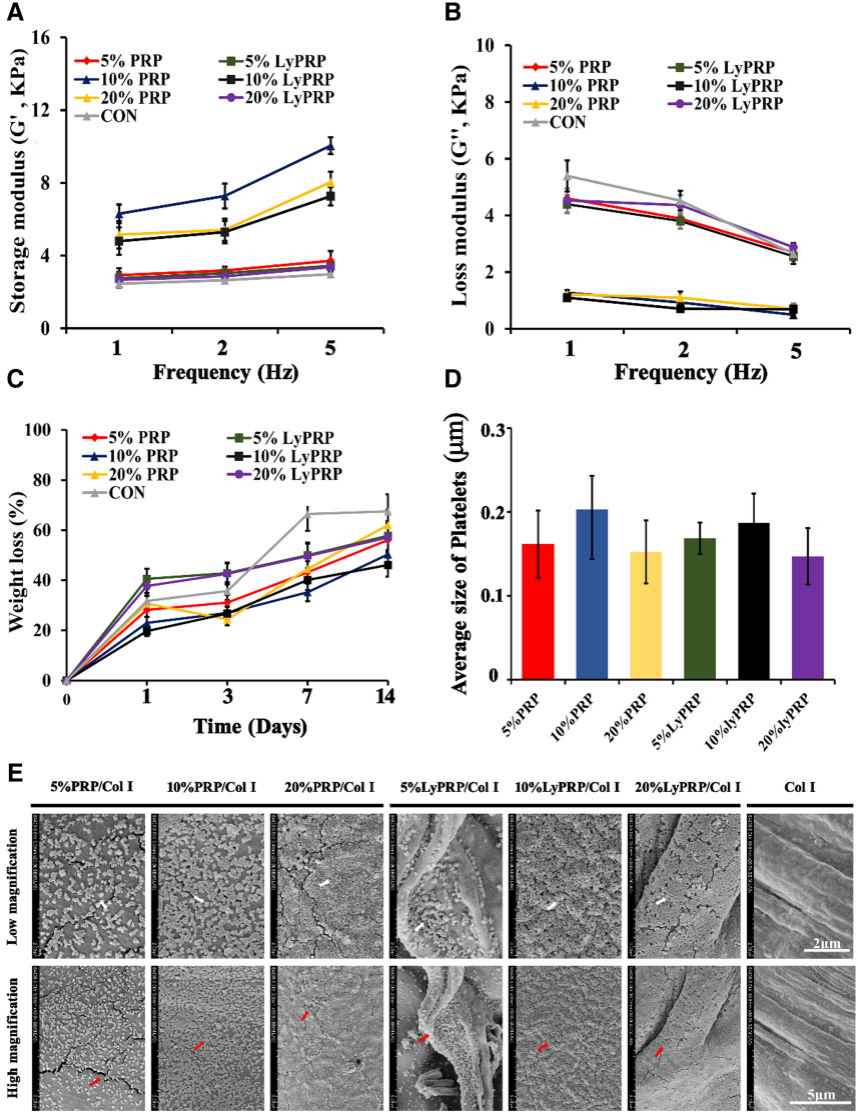
Mechanical properties of the composite materials (**A**) storage modulus (**B**) loss modulus. (**C**) Degradation behavior for the composite materials. (**D**) The pore size of different concentrations of composite materials. (**E**) SEM images of the composite materials.

Bioactive collagen gels were selected as gradual release carriers of PRP and LyPRP at different concentrations. And then, the ability of different composite hydrogels in inducing bone formation was investigated, respectively. The mechanical properties of the hydrogel scaffolds were the key factors for successful osteogenic differentiation of rBMSCs [24−26]. It can be concluded from Fig. 2A and B that, compared with the pure collagen group, the addition of PRP and LyPRP was beneficial to improve the mechanical properties of composite hydrogels. In particular, the 10% PRP/collagen and 10% LyPRP/collagen groups had the best mechanical properties. It could be concluded from the degradation curves in Fig. 2C that, compared with the pure collagen group, the addition of PRP and LyPRP at different concentrations could effectively alleviate the degradation of collagen gels. When the concentration of PRP and LyPRP was 10%, the degradation rates of composite hydrogels were relatively slow, indicating 10% was the optimal addition concentration.

Figure 2E reveals that the surfaces of collagen fibers were flat and smooth. However, the surface structures of hydrogels changed into fluffy and porous by the incorporation of PRP and LyPRP. Meantime, the visible surface roughness increased, which might be more conducive to the adhesion and spreading of cells. With PRP concentration increased, more platelets were attached to the surface of collagen fibers. It could be explained that there were some receptor proteins, such as GPVI and GPIIα receptor proteins, on the surface of platelets that could facilitate platelets adhere to collagen hydrogels [27]. Therefore, platelets were evenly distributed on the surface of collagen fibers. However, the LyPRP/Col I scaffolds exhibited stronger platelet adhesion capabilities than that of PRP/Col I, implying that platelets in LyPRP could be more effectively dispersed in collagen. It was worth noting that the quantitative analysis of SEM images by image J (Fig. 2D) showed that the size of platelets was 0.14 − 0.21 μm. The average platelets sizes of each group were 0.16, 0.20 and 0.15 μm for 5, 10 and 20% PRP/Col I, as well as 0.16, 0.18 and 0.14 μm for 5, 10 and 20% LyPRP/Col I, respectively. Among them, 10% PRP/Col I and 10% LyPRP/Col I groups showed relatively higher platelet diameters, which indicated that the concentration of 10% was beneficial to the spreading and adhesion of platelets. These results confirmed that 10% PRP/Col I and 10% LyPRP/Col I groups had obvious advantages in scaffold stability.

### Release behavior of growth factors in PRP/Col I and LyPRP/Col I composite hydrogels

**Figure 3.**
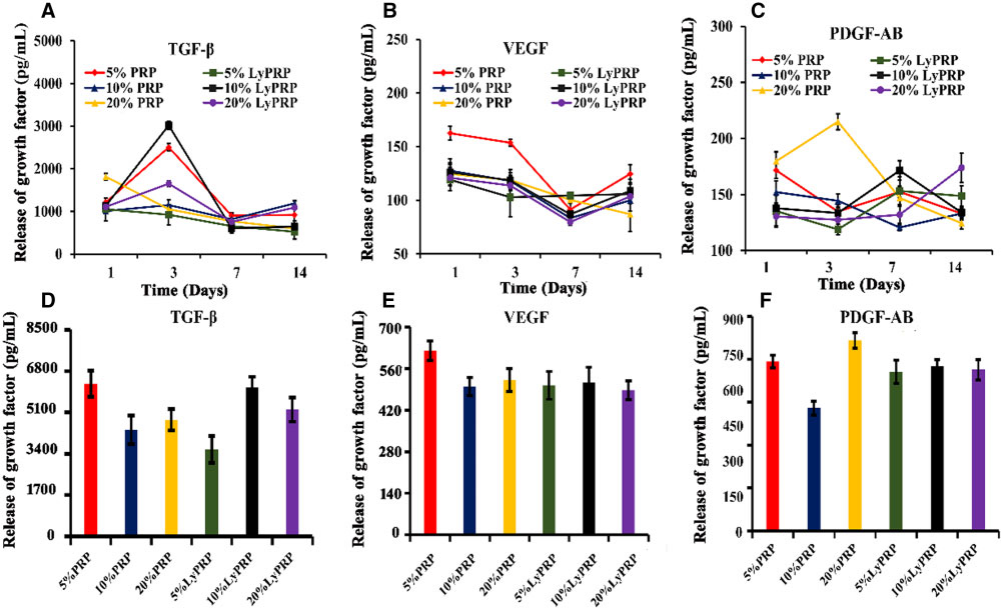
Release behavior of growth factors of the composite materials at 1, 3, 7 and 14 days. (**A**) TGF-β (**B**) VEGF. (**C**) PDGF-AB. The gross release amount of (**D**) TGF-β (**E**) VEGF. (**F**) PDGF-AB growth factors in 14 days.

In the process of bone repair, critical growth factors were required for promoting bone healing. PDGF was found as one of the earliest growth factors in platelets, which could promote the proliferation of BMSCs and wound repair [28]. TGF-β was a potential growth factor in platelets and was mainly responsible for cell differentiation and proliferation. TGF-β could promote mitosis and osteogenic differentiation of osteoprogenitor cells [29]. VEGF was a key player that regulated the recruitment, survival and activity of endothelial cells, osteoblasts and osteoclasts [30, 31]. Figure 3A−C shows that the composite gels containing different concentrations of PRP and LyPRP were soaked in culture medium to simulate the *in vitro* release behavior of these factors. From ELISA test results, it could be found that the composite hydrogels could effectively release these growth factors in PRP and LyPRP and lasted up to 14 days. Specifically, TGF-β was largely released at third day in all groups, especially in the 5% PRP/Col I group and 10% LyPRP/Col I group. After that, the release amount decreased slightly (Fig. 3A). The release of VEGF gradually decreased from Days 1 to 7 and then increased slightly on the day of 14 (Fig. 3B). The release of PDGF-AB was relatively stable during the whole process, except for the 20% PRP/Col I group, which had an obvious peak release on the third day (Fig. 3C). Compared with total release levels of VEGF and PDGF-AB, the cumulative release amount of TGF-β were several times as many as the other two groups, especially for 5% PRP/Col I group and 10% LyPRP/Col I group (Fig. 3D−F). Considering the factors release and the structural stability of composite gels, the composite collagen hydrogels with 10% PRP or LyPRP group were selected as the further studies to prove the feasibility of LyPRP in promoting bone regeneration.

### The effects of PRP/Col I and LyPRP/Col I composite hydrogels on the proliferation activity of rBMSCs

**Figure 4.**
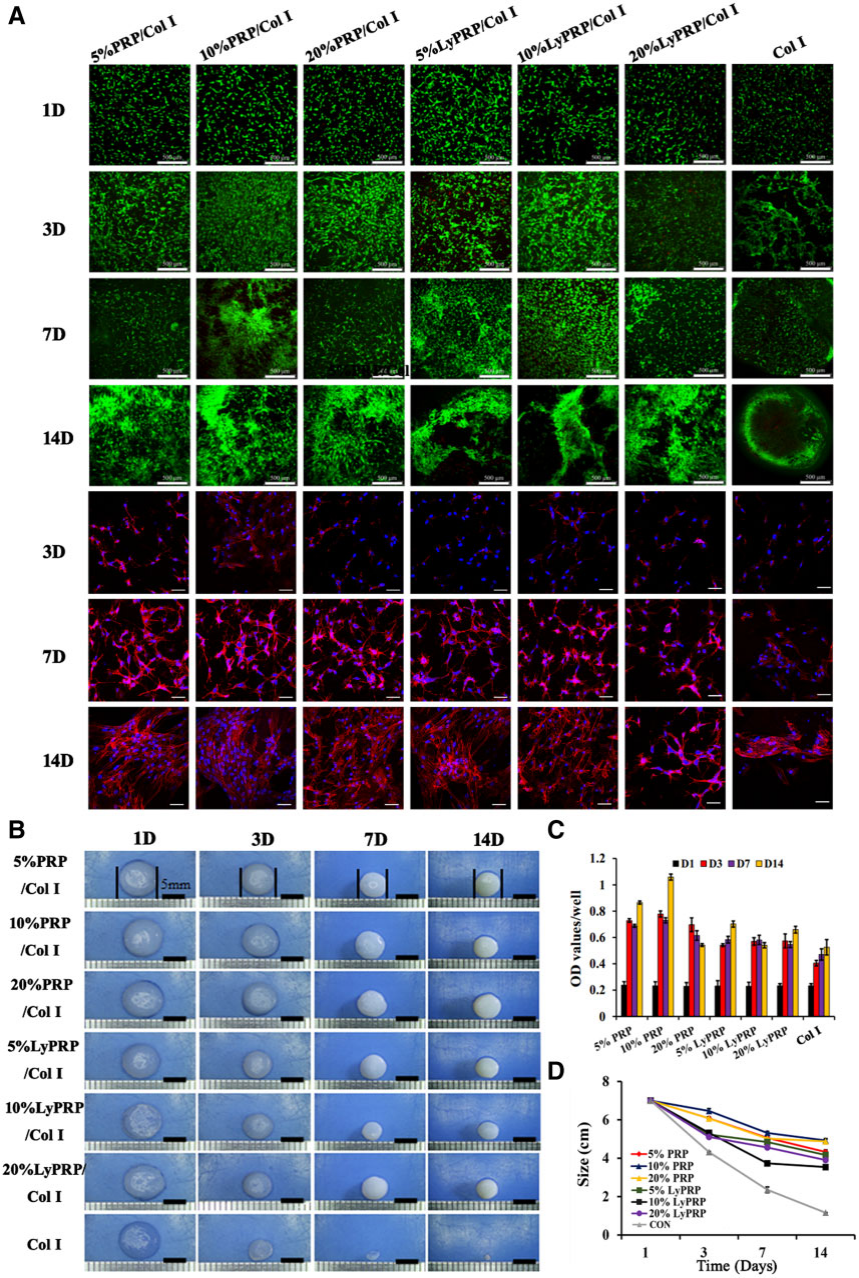
(**A**) The FDA/PI staining of 3D cultured rBMSCs with composite hydrogels at 1, 3, 7 and 14 days and the phalloidin-DAPI staining of composite hydrogels at 3, 7 and 14 days. The scale bar of FDA/PI staining images and phalloidin-DAPI staining images is 500 and 50 μm, respectively. (**B**) Macroscopic images of 3D cultured rBMSCs with hybrid hydrogels at 1, 3, 7 and 14 days. (**C**) Proliferative capacity of rBMSCs in hydrogels. (**D**) The size change of composite materials at 1, 3, 7 and 14 days.

The ideal implant materials needed degradation rate to match the cell proliferation and differentiation process so as to achieve the stable structure of regenerated tissue [32]. As shown in Fig. 4A and Supplementary Fig. S1, the distribution and proliferation behaviors of rBMSCs in PRP/Col I and LyPRP/Col I composite hydrogels were further investigated. FDA/PI staining results showed rBMSCs appeared visible growth and aggregation with the extension of the culture days. Compared with the obvious hollowing phenomenon in the pure collagen group, composite gels promoted the effective spread and growth of cells. Besides, the cell cytoskeleton in hydrogels was visualized with phalloidin-DAPI staining. The phenomenon of clusters proliferation became more obvious with the time extension, especially for 10% PRP/Col I hydrogel. With the increase of time, a larger number of rBMSCs in all hydrogels remained the normal morphology and spread out. The CCK-8 quantitative experiments showed that the number of living cells was gradually increased with the extension of culture time, except for the 20% PRP/Col I group (Fig. 4C). Cell proliferation in PRP/Col I and LyPRP/Col I hydrogels was higher than in pure collagen gels. After 14 days, the cell viability in 10% PRP/Col I hydrogels was the highest.

Corresponding visual morphological changes were shown in Fig. 4B. With the extension of culture time, the pure collagen group shrank dramatically to only 1 mm on the 14th day, which greatly restricted the spread and proliferation of rBMSCs. In experimental groups (PRP/Col I and LyPRP/Col I), the shrinkage of composite hydrogels with encapsulated PRP and LyPRP was significantly inhibited to better meet the requirements of new tissue growth space. The quantitative dimensional change curves showed (Fig. 4D) that when PRP and LyPRP were added to collagen gel and mixed with cells, the shrinkage of the hydrogels was well delayed. Therefore, the defect of excessive shrinkage of collagen was improved. Among them, the PRP/Col I groups, especially the 10% PRP/Col I group had the best effects in resisting the shrinkage of the collagen gels.

### The effects of PRP/Col I and LyPRP/Col I composite hydrogels on osteogenic differentiation of rBMSCs

**Figure 5.**
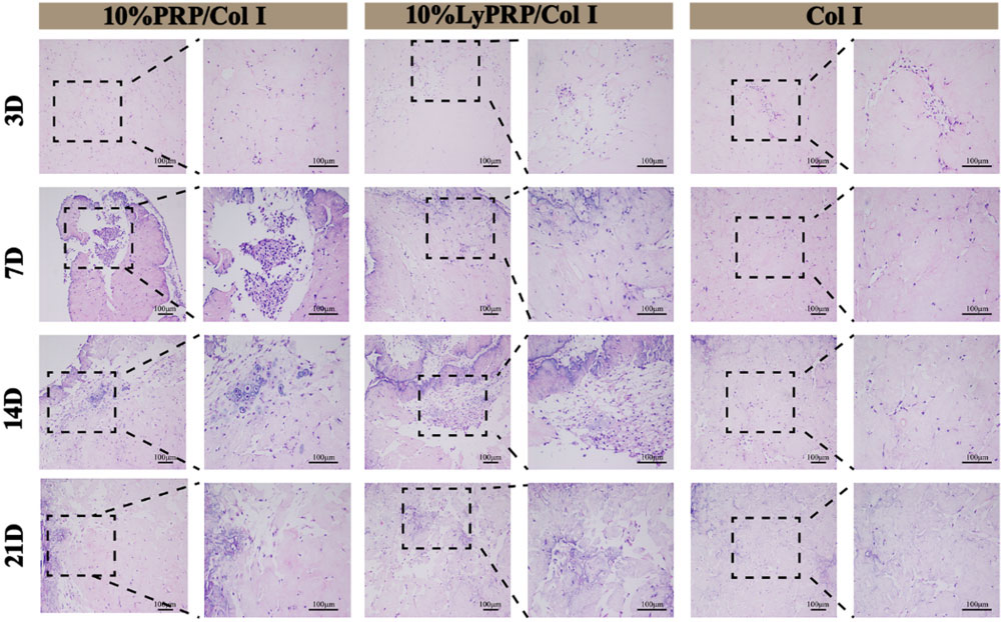
H&E staining images of cell-hydrogel constructs at 3, 7, 14 and 21 days. The dashed boxes are the corresponding high magnification images. Scale bars represent 100 μm.

MSCs as pluripotent stem cells could differentiate into osteoblasts, chondrocytes and adipocytes, which were considered as good candidates for bone therapy [33, 34]. Meanwhile, osteogenic differentiation of BMSCs was a complex process involving various growth factors and signaling pathways [35−37]. Therefore, the synergistic effect of different growth factors in LyPRP and PRP on osteogenic differentiation of rBMSCs was studied. After preliminary studies, 10% PRP/Col I and 10% LyPRP/Col I groups with better comprehensive performance were screened to investigate *in vitro* osteogenic differentiation of rBMSCs. The H&E staining images (Fig. 5) showed that evenly distributed cells appeared in all hydrogel groups. And the number of cells gradually increased with culture time extension. Compared with the pure collagen group, the 10% PRP/Col I and 10% LyPRP/Col I groups showed more cell numbers and more uniform distribution.

**Figure 6.**
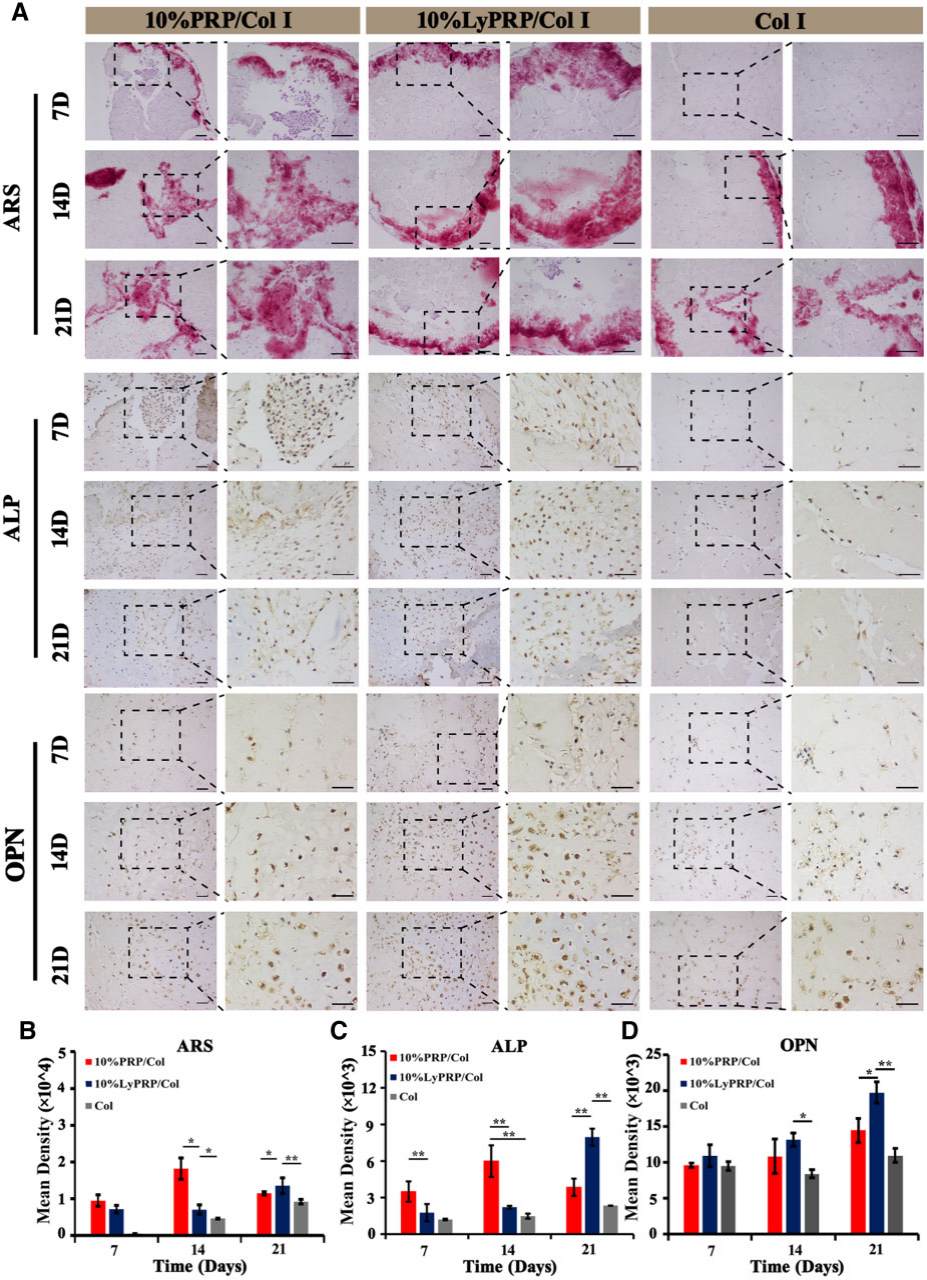
(**A**) ARS, ALP and OPN staining images of cell-hydrogel constructs at 7, 14 and 21 days. The dashed boxes are the corresponding high magnification images. Scale bars represent 100 μm. Semi-quantitative analysis of (**B**) ARS, (**C**) ALP and (**D**) OPN staining results. The data were presented as mean ± SD from three independent experiments (*n* = 3). ∗*P* < 0.05, ∗∗*P* < 0.01 and ∗∗∗*P* < 0.001.

The results of Alizarin Red staining were shown in Fig. 6A. The experimental groups (10% PRP/Col I and 10% LyPRP/Col I) secreted significantly more calcium nodules in 7 days, while almost no secretion of calcium nodules could be observed in the pure collagen group. On the 14th day, the secretion of calcium nodules in 10% PRP/Col I group was higher than that of 10% LyPRP/Col I group. After 21 days, the 10% LyPRP/Col I group presented more calcium nodules. The results were further confirmed by semi-quantitative analysis using Image J software (Fig. 6B). It could be speculated that the growth factors release of 10% PRP/Col I group mainly appeared in the early culture period, but 10% LyPRP/Col I hydrogel could continuously release in the later periods of culture and promote osteogenic differentiation of rBMSCs.

ALP was another specific IHC staining for osteogenic induction. The results of ALP staining showed that on the seventh day, some brown−black ALPs were secreted around cells in the experimental groups (10% PRP/Col I and 10% LyPRP/Col I), while almost no ALP secretion was observed in the pure group. With time raised, the amount of ALP secreted gradually increased. And the amount of ALP secreted in 10% LyPRP/Col I hydrogel was significantly higher than that of the other two groups. The semi-quantitative results by Image J software (Fig. 6C) confirmed the heaviest coloring of ALP existed in 10% LyPRP/Col I group. Analogously, OPN staining was further performed for osteogenic induction. On the seventh day, the experimental groups (10% PRP/Col I and 10% LyPRP/Col I) displayed some secreted OPN around cells, and the amount of OPN secreted gradually increased with time extension. It was worth noting that the degree of staining of OPN in LyPRP/Col I hydrogel was highest among three groups, and was confirmed by semi-quantitative results using Image J software (Fig. 6D). These obvious bone differentiation effects of rBMSCs might be attributed to the different release mechanisms of growth factors.

### Detection of specific genes expression of composite scaffolds

**Figure 7.**
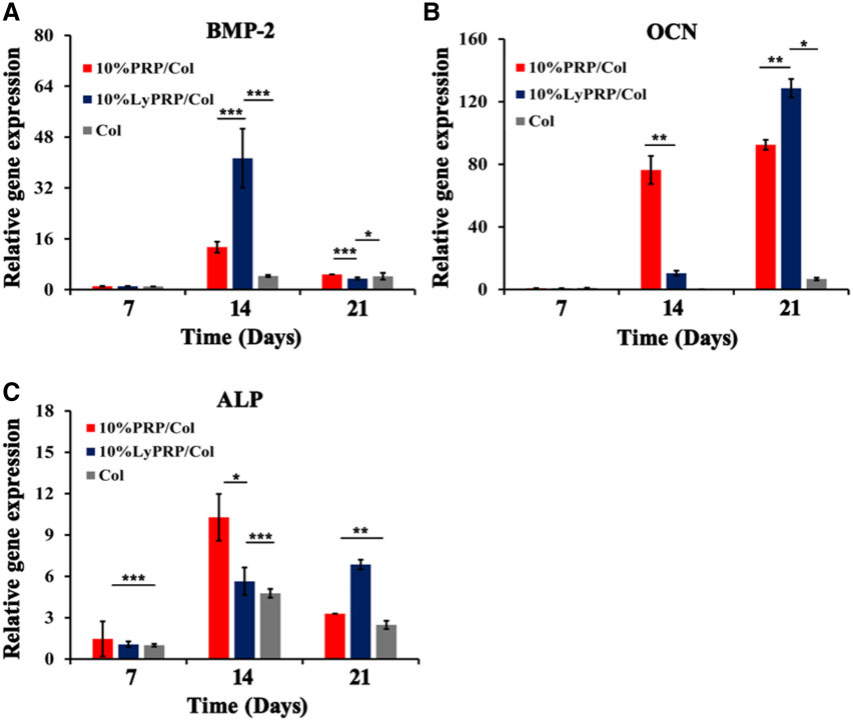
Quantitative (**A**) BMP-2, (**B**) OCN and (**C**) ALP gene expression of rBMSCs by real-time PCR analysis. The data were presented as mean ± standard deviations (SD) from three independent experiments (*n* = 3). ∗*P* < 0.05, ∗∗*P* < 0.01 and ∗∗∗*P* < 0.001.

The gene expression results of ALP, BMP2 and OCN were shown in Fig. 7. The pure collagen group showed obvious low-expression levels throughout the culture period. The gene expression results showed that 10% PRP and 10% LyPRP combined with collagen had a positive effect on osteogenic differentiation of rBMSCs. The gene expression level of BMP2 was the highest in 10% LyPRP/Col I group and presented explosive secretion at 14 days. On the other hand, the gene expression of ALP and OCN showed different time dependencies. Specifically speaking, persistent high expression appeared in 14 days for 10% PRP/Col group and 21 days for 10% LyPRP/Col group. It was speculated that the growth factors released in 10% LyPRP/Col I group would be longer-lasting, which could positively act on the entire period of cell proliferation, resulting in a significantly better osteogenic differentiation effect.

## Conclusion

Here, freeze-dried PRP (LyPRP) was encapsulated into bioactive Col I hydrogel to induce osteogenic differentiation of rBMSCs to overcome uncontrollable factors release, frequent preparation and extraction processes as well as an inconvenient form of usage of PRP and PRP/Col І composite hydrogel and pure Col І hydrogel were chosen as control. The physicochemical studies results indicated that the introduction of platelets significantly improved the mechanical properties of Col І hydrogels, and platelets were evenly distributed in Col І hydrogels networks. The sustainable release of related factors (PDGF-AB, TGF-β and VEGF) in composite hydrogels could last for more than 14 days to maintain its long-term biological activity. Further cell experiments confirmed that PRP and LyPRP could effectively alleviate collagen hydrogel *in vitro* contraction, and promote adhesion, proliferation and osteogenesis differentiation of rBMSCs. The related osteogenic gene expression results indicated that 10% LyPRP/Col І composite hydrogel could facilitate the early expression of BMP-2 and late osteogenic associated protein formation with higher expression of ALP and OCN. These results might provide new insights for the clinical application of LyPRP/Col І composite hydrogel as long-term bone repair injection.

## Supplementary data

Supplementary data are available at *REGBIO* online.

## Funding

This work was sponsored by National Key Research and Development Project of China (Grant No. 2018YFC1106800), National Nature Science Foundation of China (Grant Nos 32071352 and 81860392), Sichuan Province Scientific and Technological Achievements Transformation and Guidance Project (Grant No. 2016CZYD0004), Sichuan Province Key R&D Program (Grant No. 2019YFS0007) and Sichuan University Innovation Spark Project (Grant No. 2018SCUH0089).

*Conflict of interest statement.* None declared.

## Supplementary Material

rbaa053_Supplementary_Data
