# Supplementary material for: The effect of LyPRP/collagen composite hydrogel on osteogenic differentiation of rBMSCs
Source: Regen Biomater. 2020 Dec 11;8(1):rbaa053. doi: 10.1093/rb/rbaa053 (PMC7947583; doi:10.1093/rb/rbaa053)
Supplement: rbaa053_Supplementary_Data [file rbaa053_supplementary_data.docx]

**
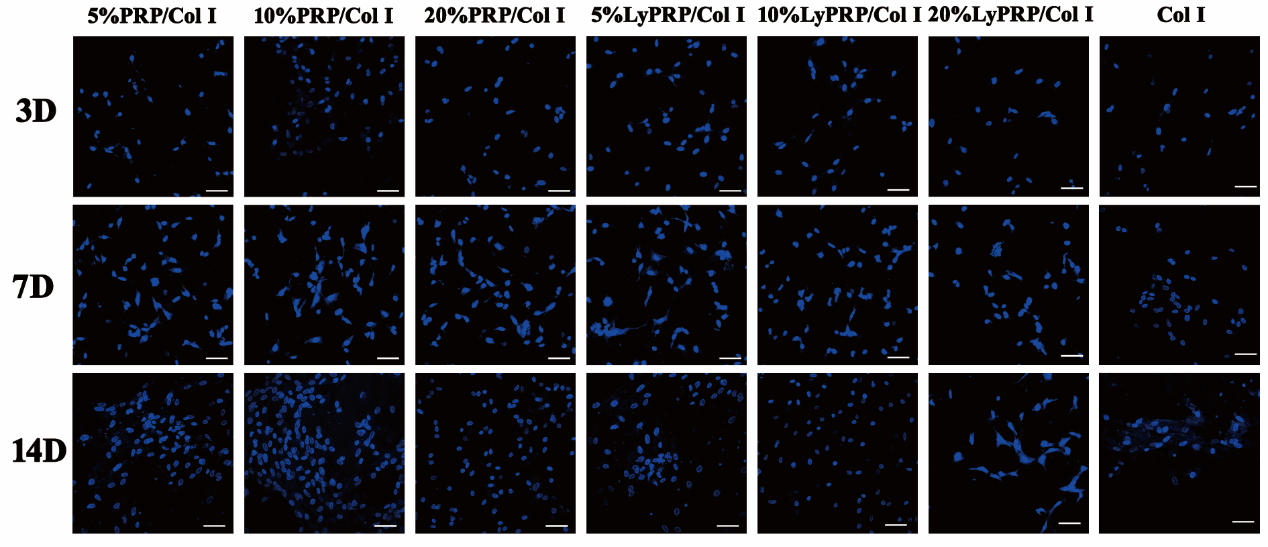
**

Figure S1. The DAPI staining of 3D cultured rBMSCs with composite hydrogels at 3,7 and 14 days. The scale bar is 50 μm.
